# The Rate of Decolorization of a Radical Ion Reagent Was Used to Determine the Phenolic Content of Various Food Extracts

**DOI:** 10.1155/2013/978968

**Published:** 2013-10-24

**Authors:** Arthur Bradley

**Affiliations:** Solar Physics Corporation, P.O. Box 548, Locust Valley, NY 11560, USA

## Abstract

Polyphenols are among the most valuable and widely studied food components. In the laboratory, they are readily extractable with aqueous alcohol. An aliquot rapidly decolorizes a measured portion of ABTS, a stable deep blue radical ion. The semilog plot of light absorption versus time is typically a straight line, and an immediately evident slope provides rapid classification in terms of gallic acid equivalents. Experimental data are presented to show general agreement with the literature. The disproportionate concentration of antioxidant in the skins and peels of fruits, vegetables, and nuts is given special attention.

## 1. Introduction

Despite our efforts to keep fit, eat sensibly, and avoid accidents, we inevitably succumb to age-related diseases. Cells cannot reproduce indefinitely, presumably due to the unavoidable accumulation of DNA errors. To achieve longevity, we are urged (among many other precautions) to avoid ionizing radiation and choose foods that neutralize free radicals. These “antioxidants” represent a new positive note in advertising. They are touted on the labels of fruit juices and green tea, and they are improving the image of dark chocolate. Cosmetic manufacturers have put these beneficial ingredients in hair conditioners and wrinkle creams.

Do antioxidants really neutralize the free radicals that kill cells and shorten life span? A skeptic might suggest that thousands of transient free radicals are present in every living cell at all times due to normal chemical activity. Nevertheless, determining which foods provide antioxidant activity, and to what extent, is an important current concern in the field of nutrition. 

A parallel development has been an increasing interest in a class of food chemicals that contain multiple hydroxyl groups on the same aromatic ring. They are referred to as polyphenols (or “phenolics”) and have gained a reputation for promoting health [1]. Polyphenols are radical scavengers and are often assumed to be responsible for antioxidant activity (AOA) in food. The theory is persuasive. When a phenolic hydrogen is removed, the extra electron would be shared around the aromatic ring—especially if there are other electrophilic substituents—and thereafter be rendered a spent force. However, not all investigators are comfortable identifying AOA with polyphenols, and some reports avoid this sideline aspect.

Without necessarily claiming that they prevent cell damage, it is still reasonable to urge the consumption of foods that neutralize free radicals (and, by extension, peroxides). As it happens, this property also makes it relatively easy to locate phenolics and gauge their concentration. Many studies have pitted them, individually or collectively, against specific diseases and conditions [2–4]. They concentrate at the surface of berries and fruits, protecting against the UV component of sunlight and fighting off alien micro-organism invasion [5]. Color in food is highly regarded as a manifestation of anthocyanins [6]. Besides vegetable pigments, the phenolics group includes several vitamins, flavonoids (catechin and quercetin), stilbenoids (resveratrol), tannic and caffeic acids, and gallate esters. 

Radical scavengers are reducing agents, and titration to completion against ferric ion (FRAP) or an organic peroxide (ORAC) can provide a quantitative assay [3]. An alternate choice is the neutralization of a solute free radical, such as DPPH (1.1-diphenyl-2-picrylhydrazyl) or ABTS (2,2′-azino-bis-(3-ethylbenzthiazoline-6-sulfonic acid), each intensely colored but rendered colorless by radical scavengers. Convenient light absorption peaks are 517 millimicrons for DPPH and 734 *μ*m for ABTS. The DPPH method was recently characterized as “slow and a little old fashioned” [7]. However, ABTS was chosen for this study largely for the convenience of dissolving and diluting the prepackaged reagent.

It is impractical to follow these ion-radical reactions to completion, and the usual procedure has been to pick an arbitrary cutoff point, typically 30 or 60 minutes. In the most sophisticated investigations, the area under a data plot was measured for comparison with a well-known standard phenolic (such as the vitamin E analog *Trolox*). The present paper describes a modification of this procedure, where measurements are taken for just the first few minutes.

Gallic acid is one of the most powerful antioxidants commonly consumed. The effective phenolic content of any foodstuff is expressed here in gallic acid equivalents (GAE). For example, fresh blueberries and strawberries are rated about one milligram of GAE per gram. Brewed coffee and tea and commercial beverages promoted as “antioxidant” generally provide between one-half and two milligrams of GAE per milliliter.

Polyphenols are famously plentiful in pomegranates and condiments like cloves and cinnamon. They are also found in red wine, cocoa, and certain fruits and nuts. The active compounds are conveniently extractable from solid foods with a mixture of ethanol and water. Beverages need only dilution to provide test samples.

An AOA “rating” is valid only for a particular specimen. Even if consistency of location, climate, and storage conditions is established, reproducibility (e.g., for mangos [8]) cannot be assured. A truly comprehensive evaluation program would include multiple specimens from various sources. The procedure described here would be useful where many parallel determinations are required.

To the vast accumulation of polyphenol and antioxidant data already in the literature, the results reported here comprise only a minor supplement. They are presented to assist in the evaluation of the simplified method.

## 2. Materials

ABTS is compounded with inert ingredients into 10 mg pellets by Thermo Scientific and sold in lots of fifty. These packets and potassium persulfate were obtained from Fisher Scientific. A model 1100 Unico spectrophotometer was purchased from GSR Technical Sales, Inc., Edmonton, AB, Canada. The unit came with a set of uniform 10 mm cuvettes for inserting liquid samples. The % light absorption scale on this student instrument extends from 000 (transparent to the incident frequency) to 1000 (essentially opaque). 

Most foods and beverages were purchased at local markets, but lingonberries and huckleberries were shipped in frozen (from Northern Europe and Washington State, resp.) by Northwest Wild Foods, Inc., Burlington, WA, USA.

## 3. Procedure

### 3.1. Reagent

A single 10 mg pellet of ABTS will dissolve in 8–10 mL of water in less than two hours at room temperature. The colorless solution begins to darken almost immediately when 0.1 mL of 0.1 N aqueous potassium persulfate is added. Color continues deepening for another six hours, after which the radical ion is diluted with 95% ethanol and stored in a refrigerator. Note: the persulfate gradually loses oxidizing power and should be renewed after six months. 

To make it ready for use, the deep blue ABTS concentrate was further diluted with 95% ethanol. Every test started with the reagent concentration adjusted to provide (relative) light absorption between 800 and 900 (*A*_0_) at 735 millimicrons on the Unico dial. 

### 3.2. Preparation of Test Samples

Nutmeats, vegetables, and fruit pulps were chopped into small fragments. When appropriate, the subject item was peeled and the latter cut into small pieces. Samples were weighed into 125 mL Erlenmeyer flasks, and a measured volume of 60% alcohol was added to extract the phenolic content. The flasks were labeled, stoppered, and stored at room temperature, with occasional agitation. The initial assay after two days was often repeated the next day to show that the antioxidant level in the extract was no longer increasing.

### 3.3. Testing

**Figure 1:**
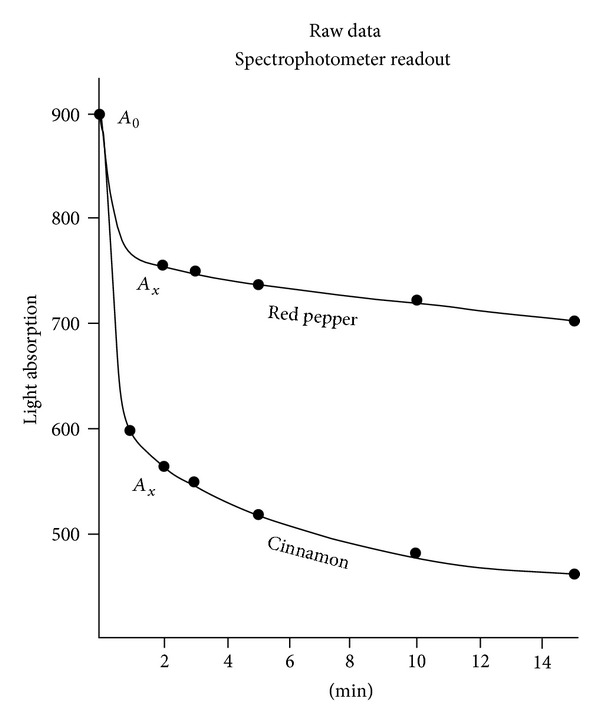


The extract of a foodstuff with little or no antioxidant activity could be examined directly. However, it was often necessary to dilute this solution further with water or alcohol to slow ABTS decolorization to a measurable rate. Timing started when a small aliquot of this dilution was shaken with 3.0 mL of the reagent. The “absorption” readout (*A*_*x*_) dropped rapidly at first and then slowly (see Figure 1). Note: the ideal sample dilution would result in between one and two micrograms of GAE in a 0.10 mL aliquot.

### 3.4. Example

A portion of powdered cinnamon (brand A) weighing 2.2 g was immersed in 40 mL of 60% ethanol. After three days, with occasional agitation, 1.00 mL was removed and diluted to 70 mL with water. A  .050 mL aliquot of the 70 : 1 dilution reduced the ABTS absorption readout from 900 (*A*_0_) to 601 (*A*_*x*_) in one minute, to 564 in two minutes, 544 in three minutes, 517 in five minutes, 479 in ten minutes, and 460 in fifteen minutes. These data points, plus a comparable set for crushed red pepper (which also happens to start at 900), are presented in Figure 1.

**Figure 2:**
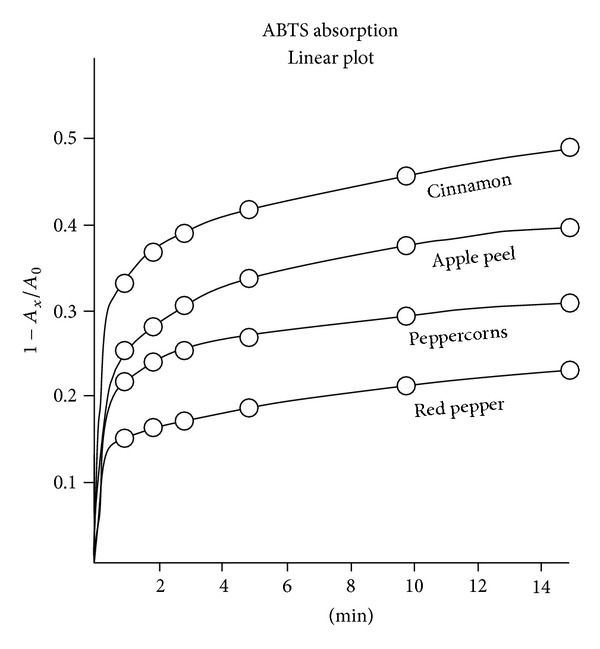


**Figure 3:**
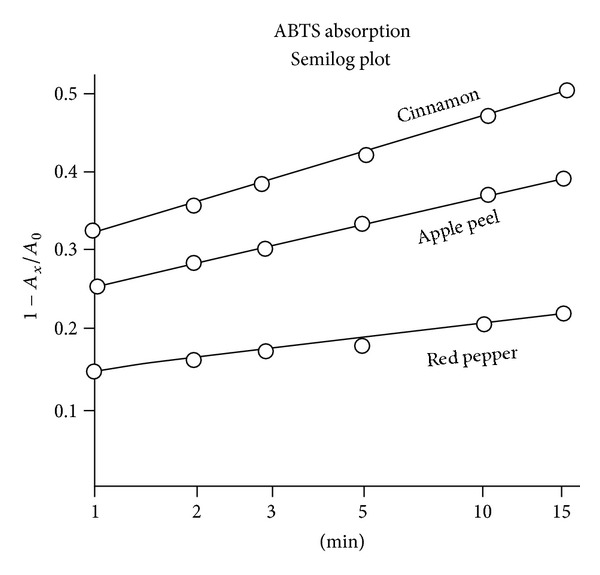


The fractional amount of ABTS consumed at each interval was 1 − *A*_*x*_/*A*_0_. These values for cinnamon and red pepper and two other test specimens are plotted as a linear function of time in Figure 2 and on a semilog scale in Figure 3.

**Figure 4:**
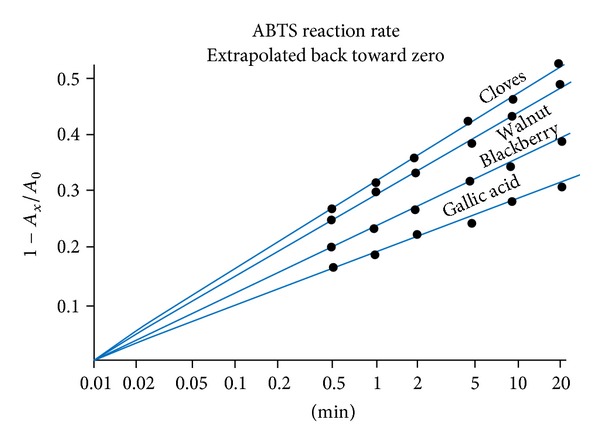


**Figure 5:**
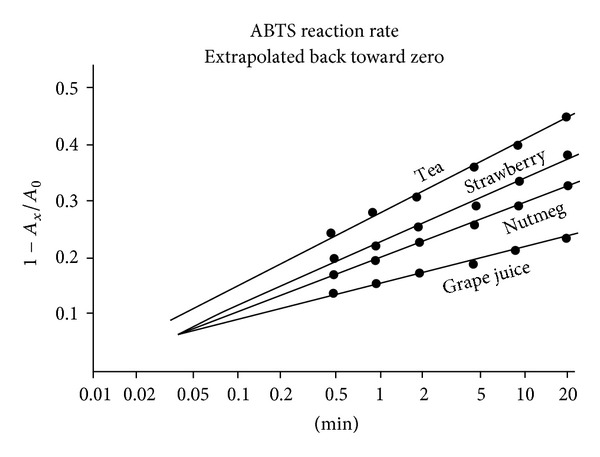


Since a reliable 30 second point could also be obtained, extra cycles were added to the plot to extrapolate back toward zero. Absorption data for cloves, walnut, and blackberry, plus gallic acid, are presented in Figure 4. Similar data for tea, strawberry, nutmeg, and grape juice are assembled in Figure 5. It should be pointed out that the charts offer no clues to AOA ratings. Each assay had its own individual sample weight and solvent volume (Table 1).

**Figure 6:**
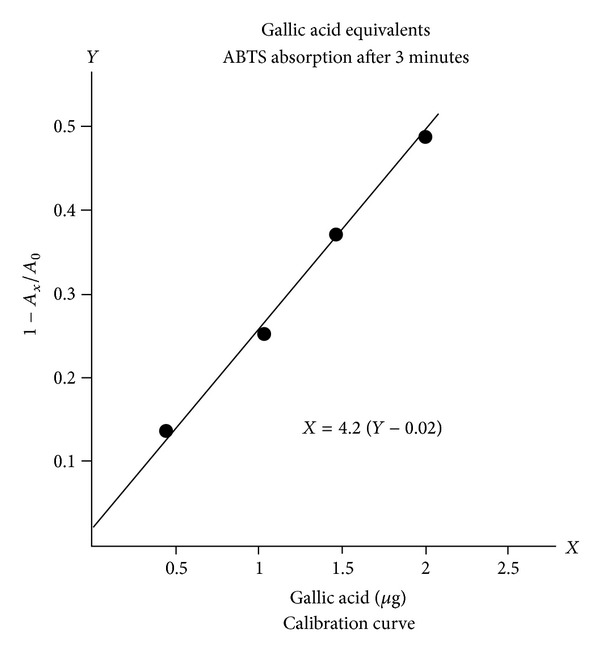


Figure 6 presents the gallic acid reduction of ABTS absorption (after three minutes) as a function of its concentration. This is the calibration curve.

### 3.5. Sample Analysis

Conversion of the raw data for cinnamon (Figures 1, 2, and 3) to a meaningful criterion of AOA is described here. Reduced absorption is represented on the *y*-axis by the dimensionless number 1 − *A*_*x*_/*A*_0_. In this investigation, light absorption after three minutes was chosen to represent the relative potency of each individual foodstuff as well as the standard.

After three minutes we have the following:

 
$1-\frac{A_{x}}{A_{0}}\;=\;1-\frac{544}{900}\;=\;0.40$.

On the calibration chart (Figure 6), this corresponds to 1.60 micrograms of gallic acid in .05 mL or 32 *μ*g/mL in the 70 mL of diluted test sample. The latter would therefore have contained a total of 2.24 mg, expanding to 90 mg in the original 40 mL of solvent utilized for extraction. This 90 mg (of gallic acid equivalent) had been obtained from 2.2 grams of powdered cinnamon, which is therefore assigned a phenolic rating of 90/2.2 or 41 mgGAE/gram. A strong performance, but still only 4.1% of the radical scavenging benefit of pure molecular gallic acid. Gallic acid would rate 1000 mgGAE/gram. Cinnamon, at 41 mgGAE/gram, has just 4.1% of the AOA potency of gallic acid.

The three-minute point is late enough to minimize the slight uncertainty in the exact starting time, but before any drift in the spectrophotometer or autodecomposition of the reagent is likely to take effect. The key measurement comes early, but it is advisable to take additional points occasionally to confirm the linear response.

## 4. Discussion

Radical scavenging in food and drink has long been identified with the “phenolic” content—moderately sized molecules with clusters of aromatic hydroxyl groups [9]. These entities are readily extractable with polar solvents [10, 11]. Their reaction with the ABTS radical ion is quite rapid, with a typical “half-life” (as measured by ABTS concentration) of about one minute [12]. A plot of decreased light absorption against the logarithm of time was almost invariably a straight line.

As mentioned above, several investigative teams have evaluated AOA by measuring the area under an absorption curve (e.g., Figure 2) and evaluating it by a comparison with a commercial vitamin E analog [4, 11]. In a recent publication, Barton noted that first order kinetics can be extrapolated back to zero time and pointed out that this method is independent of sample concentration, but he continued to assign AOA values in terms of *Trolox* equivalents [13]. With linear data plots like in Figures 3, 4, and 5, this elaboration appears unnecessary.

Since it is impractical and unnecessary to identify the active molecules in every food sample, many authors have expressed their results in gallic acid equivalents [7, 8, 14]. Absorption data extrapolate back to a starting point before one second. Several three-minute points for gallic acid generated the calibration curve and conversion formula of Figure 6.

## 5. Procedure Illustrated

Typical examples of low to moderate dietary AOA are presented for various berries and some other food groups in Table 1, which is constructed to show how raw data is converted to mgGAE/g. The calibration curve (Figure 6) derived micrograms of GAE in the aliquot from the absorption fraction 1 − *A*_*x*_/*A*_0_. Four parallel determinations with dry lingonberries are included as examples of the reproducibility of the method. It will be observed that most fruit pulps and vegetables had considerably less than one milligram of gallic acid equivalent per gram (with pomegranate a notable exception).

Phenolics are part of the defense strategy of plant life against invasive species. They concentrate on the surface. Łata and Tomala reported that the phenolic content in the peel of a Granny Smith apple was three times that found for the whole fruit, including the peel [5]. When the peel is separated from the pulp, the contrast is even more striking. Where skin is very thin, as on peanut or chestnut, the skin/pulp AOA ratio can be two orders of magnitude.  Table 2 compares pulps with peels.

The extraordinary concentration of phenolics in peanut skins had been observed by Ballard et al. [14]. Tsujita et al. recently characterized the high phenolic content of the thin skin on chestnut meat [15].

## Figures and Tables

**Table 1:** Procedure illustrated, aliquots 0.10 mL.

Source of AOA	Sample grams	^ @^mL	^ #^Dilution	1 − *A*_*x*_/*A*_*o*_	GAE ugms	Calculation	mgGAE per gm
Cloves (A)	1.2	51	1 : 200	0.29	1.13	×2000 × 51/1.2	96—
Cloves (B)	1.4	54	1 : 240	0.26	1.01	×2400 × 54/1.4	93—
Cinnamon (B)	1.6	52	1 : 120	0.28	1.09	×1200 × 52/1.6	43—
Oregano	1.5	48	1 : 60	0.235	0.99	×600 × 48/1.5	19—
Marjoram	2.5	47	1 : 70	0.30	1.18	×700 × 47/2.5	15—
Cocoa	2.6	48	1 : 40	0.295	1.16	×400 × 48/2.6	8.5
Thyme	1.4	47	1 : 20	0.275	1.07	×200 × 47/1.4	7.2
Nutmeg	1.5	48	1 : 10	0.39	1.55	×100 × 48/1.5	5.0
Black currants	4.0	43	1 : 30	0.29	1.08	×300 × 43/4.0	3.5
Cumin (A)	2.4	45	1 : 15	0.29	1.13	×150 × 45/2.4	3.2
“Cumino” (B)	1.8	48	1 : 12	0.26	1.01	×120 × 48/1.8	3.2
Huckleberries	3.9	46	1 : 15	0.28	1.09	×150 × 46/3.9	1.9
Pepper (ground)	3.8	46	1 : 10	0.30	1.12	×100 × 46/3.8	1.3
Blueberries	4.2	53	1 : 10	0.26	0.96	×100 × 53/4.2	1.2
Sunflower seeds	4.7	42	1 : 10	0.325	1.22	×100 × 42/4.7	1.1
Strawberries	9.5	58	1 : 12	0.36	1.43	×120 × 58/9.5	1.05
Lingonberries I	2.4	43	1 : 5	0.28	1.09	×50 × 43/2.4	0.98
Lingonberries II	2.6	41	1 : 5	0.33	1.30	×50 × 41/2.6	1.03
Lingonberries III	2.4	41	1 : 5	0.30	1.18	×50 × 41/2.4	1.00
Lingonberries IIII	2.5	42	1 : 5	0.31	1.22	×50 × 42/2.5	1.02
Blackberries	4.9	51	1 : 8	0.26	0.96	×80 × 51/4.9	1.00
Flax seed	3.2	43	1 : 5	0.31	1.16	×50 × 43/3.2	0.82
Collard greens	4.7	47	1 : 5	0.24	0.88	×50 × 47/4.7	0.44
Parsley	4.2	48	1 : 3	0.28	1.09	×30 × 48/4.2	0.37
Cocoa puffs	4.2	42	1 : 3	0.29	1.13	×30 × 42/4.2	0.34
Red pepper	3.0	44	—	0.365	1.45	×10 × 44/3.0	0.21
Wheat germ	3.8	53	—	0.34	1.34	×10 × 53/3.8	0.19
Banana	5.9	45	—	0.42	1.60	×10 × 45/5.9	0.12

^@^Extraction solvent was 60% ethanol.

^#^Water dilution before testing.

GAE: gallic acid equivalents.

**Table 2:** Pulps and peels.

Foodstuff source of AOA	Pulp—Peel		
	mgGAE/gram		
Pomegranate	−23*	35—	
Delicious apple	0.50	−3.8	Red
Mango	0.47	−8.0	Red
Mango	0.46	−4.3	Green
Granny Smith apple	0.38	1.15	Green
Apricot	0.31	−1.2	
Chestnut	0.30	33—	Skin
Black plum	0.25	−3.9	
Pearl onion	0.20	1.25	
Eggplant	0.18	−1.6	Rind
Peanut	0.12	27—	Skin
Acorn squash	0.12	0.60	
Bose pear	0.10	0.97	
Red onion	0.09	0.86	Skin
Rhubarb	0.08	−1.5	
Italian pepper	0.07	0.43	Green
Peach	0.065	0.23	
Tangerine	0.06	0.37	Rind
Sweet potato	0.055	0.32	
Garlic, raw	0.05	0.31	
Northwest pear	0.045	1.00	Red
Parsnips	0.03	0.27	
Radish	0.03	0.45	Red
Red potato	0.03	0.34	

The pulp (or “membrane”) of pomegranate is not considered edible. Its high polyphenol content is not lost, however, since most pomegranates are crushed whole (with seeds) to recover the juice.

## References

[1] Scalbert A, Johnson IT, Saltmarsh M (2005). Polyphenols: antioxidants and beyond. *The American journal of clinical nutrition*.

[2] Lee J, Koo N, Min DB (2004). Reactive oxygen species, aging, and antioxidative nutraceuticals. *Comprehensive Reviews in Food Science and Food Safety*.

[3] Moon J-K, Shibamoto T (2009). Antioxidant assays for plant and food components. *Journal of Agricultural and Food Chemistry*.

[4] Leo L, Leone A, Longo C, Lombardi DA, Raimo F, Zacheo G (2008). Antioxidant compounds and antioxidant activity in early potatoes. *Journal of Agricultural and Food Chemistry*.

[5] Łata B, Tomala K (2007). Apple peel as a contributor to whole fruit quantity of potentially healthful bioactive compounds. Cultivar and year implication. *Journal of Agricultural and Food Chemistry*.

[6] Joseph JA, Nadeau DA, Underwood A (2002). *Color Code*.

[7] Jimenez-Alvarez D, Giuffrida F, Vanrobaeys F (2008). High-throughput methods to assess lipophilic and hydrophilic antioxidant capacity of food extracts in vitro. *Journal of Agricultural and Food Chemistry*.

[8] Manthey JA, Penelope P-V (2009). Total phenols, *in vitro* antioxidant capacity, and phenolic profiles of five varieties of mango. *Journal of Agricultural and Food Chemistry*.

[9] Solomon A, Golubowicz S, Yablowicz Z (2006). Antioxidant activities and anthocyanin content of fresh fruits of common fig. *Journal of Agricultural and Food Chemistry*.

[10] Wu X, Gu L, Holden J (2004). Development of a database for total antioxidant capacity in foods: a preliminary study. *Journal of Food Composition and Analysis*.

[11] Xie Z, Liu W, Huang H (2010). Chemical composition of five commercial gynostemma pentaphyllum samples and their radical scavenging, antiproliferative, and anti-inflammatory properties. *Journal of Agricultural and Food Chemistry*.

[12] Walker RB, Everette JD (2009). Comparative reaction rates of various antioxidants with ABTS radical cation. *Journal of Agricultural and Food Chemistry*.

[13] Barton HJ (2010). Standardization of methods for the estimation of total antioxidant activity by the use of extrapolation to zero sample concentration. A novel standard. 1. ABTS cation radical scavenging. *Journal of Agricultural and Food Chemistry*.

[14] Ballard TS, Mallikarjunan P, Zhou K, O’keefe SF (2009). Optimizing the extraction of phenolic antioxidants from peanut skins using response surface methodology. *Journal of Agricultural and Food Chemistry*.

[15] Tsujita T, Yamada M, Takaku T, Shintani T, Teramoto K, Sato T (2011). Purification and characterization of polyphenols from chestnut astringent skin. *Journal of Agricultural and Food Chemistry*.

